# Research funding for addressing tobacco-related disease: an analysis of UK investment between 2008 and 2012

**DOI:** 10.1136/bmjopen-2016-011609

**Published:** 2016-07-04

**Authors:** Mary Hall, Ilze Bogdanovica, John Britton

**Affiliations:** 1 StR Public Health, Public Health Department, LCC, Leicester, UK; 2 UK Centre for Tobacco Control Studies, Division of Epidemiology and Public Health University of Nottingham, Nottingham, UK

**Keywords:** Research Funding, Prevention, Tobacco related disease

## Abstract

**Introduction:**

Tobacco use is the leading cause of preventable death in the UK. However, research spending on tobacco-related disease, and particularly smoking prevention, is thought to be low. We therefore aimed to assess the relation between tobacco-related research investment and disease burden from 2008 to 2012.

**Methods:**

We used the Health Research Classification System to classify UK government and charitable research funding by broad health category and then by tobacco prevention research and 18 WHO defined tobacco-related diseases. We used UK mortality figures to calculate disease-specific tobacco attributable deaths and then compared disease specific and tobacco prevention research investment with all cause and tobacco attributable mortality over the 5-year period and as annual averages.

**Results:**

12 922 research grants were identified with a total value of £6.69bn, an annual average of £1.34bn. Annually an average of 110 000 people die from tobacco-related disease, approximately 20% of total deaths. £130m is invested in researching tobacco-related disease each year and £5m on tobacco prevention, 10.8% and 0.42% of total annual research funding, respectively. Prevention research equated to an annual average of £46 per tobacco attributable death or one pound for every £29 spent on tobacco-related disease. Funding varied widely for diseases with different numbers of deaths (eg, lung cancer £68 per all cause death, cervical cancer £2500), similar numbers of deaths (leukaemia £983 per death, stomach cancer £43) or similar numbers of tobacco attributable deaths (eg, colorectal cancer £5k, pancreatic cancer £670, bladder cancer £340).

**Conclusions:**

Tobacco-related research funding is not related to burden of disease or level of risk. As a result certain diseases receive a disproportionately low level of research funding and disease prevention funding is even lower.

**Strengths and limitations of this study**

- Analysis of research funding was comprehensive and over a 5-year period allowing for differences in annual funding trends. However, it is likely that some funding sources were missed; commercial and industry funding was not included since, to the best of our knowledge, it does not influence national research policy.
- The study used internationally recognised methods of categorising research funding and calculating numbers of tobacco attributable death; use of the smoking impact ratio may have over-estimated the number of tobacco attributable deaths.
- Using mortality as a measure of disease burden is simple and unequivocal but excludes impact of time spent ill or age of death.
- Methods used were simple and easily replicable across research disciplines.

## Introduction

Tobacco use is the leading cause of preventable death in the UK, killing ∼100 000 people every year.^1^ Half of all smokers die prematurely as a consequence of their smoking unless they quit,^2^ and their death is often preceded by several years of ill health.^3^ Smoking is most prevalent among the most disadvantaged in society,^4^ ^5^ and is the largest avoidable cause of social inequalities in health and life expectancy.^6^ ^7^ Wider society is impoverished by the healthcare and wider societal costs of smoking^8–11^ and smoking contributes significantly to levels of poverty in the UK.^12^ Since smoking is entirely avoidable, preventing smoking is the most effective way to improve health and well-being in the UK. Supporting research to improve smoking cessation and prevent uptake of smoking should therefore be a high priority for research funders.

The UK government has recommended that health research priorities should be based on the country's health needs and priorities, which are set in relation to the impact of disease and illness.^13^ However researchers across a range of disciplines have claimed that their specific research area is underfunded^14–16^ and have used a variety of disease impact or burden measures to support these claims.^14^ ^17–19^ The huge detrimental contribution of tobacco to the UK's health and economy would suggest that tobacco smoking is a priority for research investment, but evidence to date on whether this is actually the case in practice is lacking. This study therefore aimed to assess the relation between investment in UK health research and disease burden, with a particular focus on tobacco research and burden of tobacco-related disease.

## Methods

Disease burden is usually estimated using one or a combination of three measures: mortality, morbidity and the impact of disease on the economy. For this study we used mortality from 18 tobacco attributable diseases as identified by the WHO.^20^ We obtained numbers of total and disease specific deaths for the period 2008–2012, broken down by age and sex, from the Office of National Statistics for England and Wales (ONS), the General Register Office for Scotland, and the Northern Ireland Statistics and Research Agency.^21–23^ Diseases were defined using the International Classification of Diseases and Related Health Problems 10th Revision (ICD-10).^24^ Deaths on breast and prostate cancer were collected for comparison purposes from the same sources.

Estimates of smoking prevalence by age and gender were calculated for each year for the same period using the smoking impact ratio (SIR), whereby deaths from lung cancer are used as a proxy measure for smoking prevalence.^25^ Estimates of age and sex-specific tobacco-related relative risks (RR) were taken from updated Cancer Prevention Study (CPS-II) figures.^26^ Disease, age and sex-specific population attributable fractions were then calculated using the RR, the SIR and the total number of deaths for each disease. These were combined to establish disease specific total deaths attributable to tobacco and an overall total number of deaths attributable to tobacco each year.

### Research funding

Of the £3 billion of public funds provided annually for medical research in the UK, one-third comes from the National Institute for Health Research (NIHR), one-third from the Medical Research Council (MRC) and one-third from charitable bodies.^27^ We therefore obtained data on UK government health research funding for the five years from 2008 to 2012 from NIHR (England), the Chief Scientist's Office (CSO, Scotland), the National Institute for Social Care and Health Research (NISCHR, Wales) and the Health and Social Care Research Department (HSCRD, Northern Ireland); and directly from the MRC. Charitable research funding is provided by an extensive range of organisations but 85% of funding is provided by five charities: Arthritis Research Campaign (ARC), Cancer Research UK (CRUK), Leukaemia and Lymphoma Association, the Wellcome Trust and British Heart Foundation (BHF).^27^ Since arthritis and rheumatism primarily cause disability but not death we excluded the ARC, but sought to obtain data on research funding allocated between 2008 and 2012 from the other above named charities. The Leukaemia and Lympohoma Association declined to provide funding information so their grants are not included in the analysis. Data were obtained either from organisational websites if available and otherwise by direct contact with the funding organisation.

The 12 922 grants identified by the eight funders (the Wellcome Trust, CRUK, BHF, MRC, NIHR, NISCHR, CSO and HSCRD) were classified by the lead author using grant titles and the UK Health Research Classification System (HRCS)^28^ with the exception of MRC and NIHR who routinely classify their own grants using the HRCS (3796 grants in total). Grants were allocated to up to five of the following 21 health category labels: blood, cancer, cardiovascular, congenital disorders, ear, eye, infection, inflammatory and immune system, injuries and accidents, mental health, metabolic and endocrine, musculoskeletal, neurological, oral and gastrointestinal, renal and urogenital, reproductive health and childbirth, respiratory, skin, stroke, generic health relevance, other. Categories of grants covering more than one health area were equally apportioned depending on the number of areas covered. For example two allocated categories in one grant would be equally apportioned 50%. Grants covering more than five areas were categorised as generic health relevance. Grants that did not fit into any category were classified as other, such as PhD posts that did not specify an area of research.

Grant funding was adjusted to 2012 prices using the UK HM Treasury Inflator Calculator^29^ and because information regarding length of funding awards was not consistently available, funding calculations were based on the year of award. Total annual category-specific funding was calculated by multiplying the category apportioned percentage for each grant by the total grant award and combining the resulting figures for each category. Average annual funding per category was then calculated.

Non-relevant grants were then excluded from the final analysis. These included grants awarded to overseas institutes, those solely for overseas research or those for the arts, journalism, veterinarian or medical humanities research. A full list of excluded grants is available on request. To ensure that no relevant grants had been excluded, a search was run on all excluded grant titles for the words ‘tobacco’, ‘smoking’ or ‘nicotine’; none were identified. Included grant titles (10 647 in total for the 5-year study period) were further analysed for the 18 tobacco-related disease areas identified by WHO^20^ and breast and prostate cancer for comparison purposes. As before, equal percentages were applied where there were two or more disease areas in any particular grant title. An additional category of ‘tobacco’ was created to estimate direct investment in tobacco prevention research and included all grants that contained ‘tobacco’, ‘smoking’ or ‘nicotine’ in their titles. All grant titles identified in this manner were checked for relevance to tobacco prevention. Total annual disease-specific funding was calculated by multiplying the disease-specific apportioned percentage for each grant by the total grant award. Average annual funding per disease area and for tobacco was then calculated.

### Analysis

Descriptive analysis was carried out exploring all-cause deaths, deaths attributable to tobacco and annual average funding by health category, tobacco-related disease and tobacco prevention research. Funding related to broad HRCS categories included all research grant funding (12 922 grants) in order to provide an overview of total funding. Funding related to tobacco prevention and disease research excluded non-relevant grants as per the exclusion criteria (10 647 grants in total). We calculated disease-specific annual average funding as a proportion of total annual average funding and used linear regression analysis to assess the association between proportion of funding and number of all-cause and tobacco-related deaths. We further calculated the annual average research spend per all-cause and tobacco attributable death for each tobacco-related disease by dividing the annual average funding for that disease by its annual average number of all-cause and then, tobacco attributable deaths. All analysis including regression analysis was carried out using Microsoft Excel 2010.

## Results

### Deaths from tobacco-related diseases

**Figure 1:**
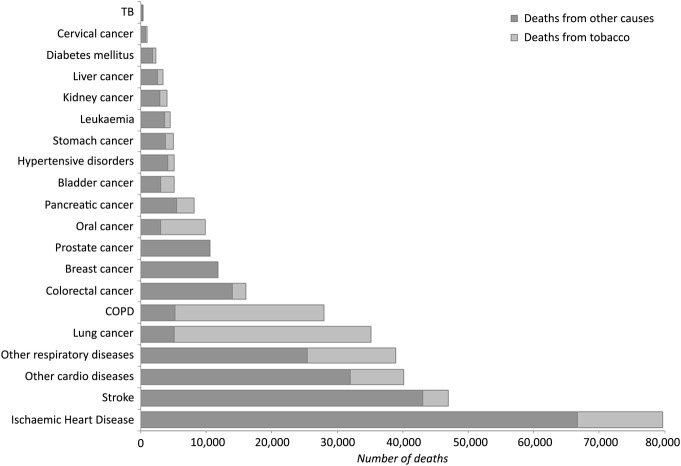
Disease-specific deaths: tobacco and all cause. COPD, chronic obstructive pulmonary disease; TB, tuberculosis.

Across 2008–2012 an annual average of 110 500 deaths were estimated to be attributable to tobacco, equating to 20% of all UK deaths per year. From the tobacco-related diseases considered, ischaemic heart disease (IHD) was responsible for the most deaths from all causes, but lung cancer and chronic obstructive pulmonary disease (COPD) contributed the most deaths from tobacco (figure 1).

### Research funding

#### Funding by health category

Total research funding for all health categories over the 5 years amounted to £6.69bn, equivalent to an annual average of £1.34bn. Further information regarding specific funding by health category is available in online supplementary figure S1.

10.1136/bmjopen-2016-011609.supp1 Supplementary figure

#### Funding by tobacco prevention and tobacco-related disease

**Figure 2:**
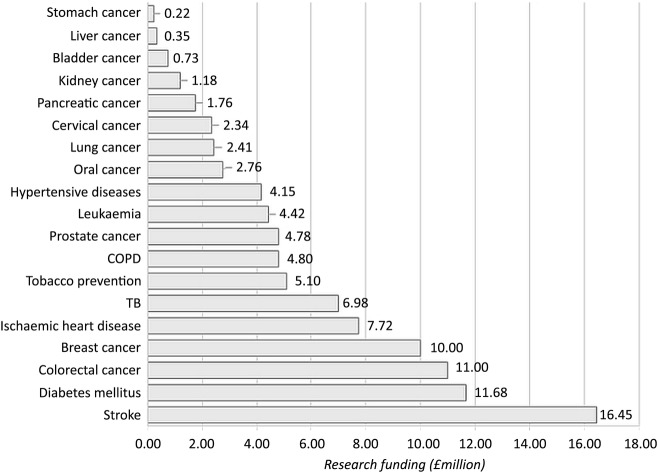
Annual average funding by tobacco-related disease. (Funding awarded by the Leukaemia and Lymphoma Association is not included in this analysis so funding for leukaemia is likely to be higher than the figure given). COPD, chronic obstructive pulmonary disease; IHD, ischaemic heart disease; TB, tuberculosis.

Following application of the exclusion criteria on non-relevant grants, total research funding (of the remaining 10 647 grants) amounted to £6.05bn over the 5 years or an annual average of £1.21bn. Total funding for diseases identified as tobacco-related amounted to an annual average of £130m or 10.8% of the total annual average funding of £1.21bn (figure 2). Of all WHO tobacco-related diseases, stroke received the largest quantity of funding (£16.5m per year or 1.4% of total annual average funding) followed by diabetes mellitus (£11.7m, 1% of total) and colorectal cancer (£11m per year). Research into stomach, liver and bladder cancer each amounted to <£1m per year respectively or <0.1% of total annual average funding. In particular, stomach and liver cancer each received an average of <£400k in research funding per year.

There were 78 grants including ‘smoking’, ‘tobacco’ or ‘nicotine’ in the grant title, of which two were related to specific treatment for tobacco-related disease and so were classified under their specific disease. The remaining 76 all involved smoking prevention activities including policy research or infrastructure support, and were thus categorised as ‘tobacco prevention’.

An average of £5.1m was spent each year on tobacco prevention research, or 0.42% of the total annual average funding of £1.21bn.

### Relation between research investment and mortality

**Figure 3:**
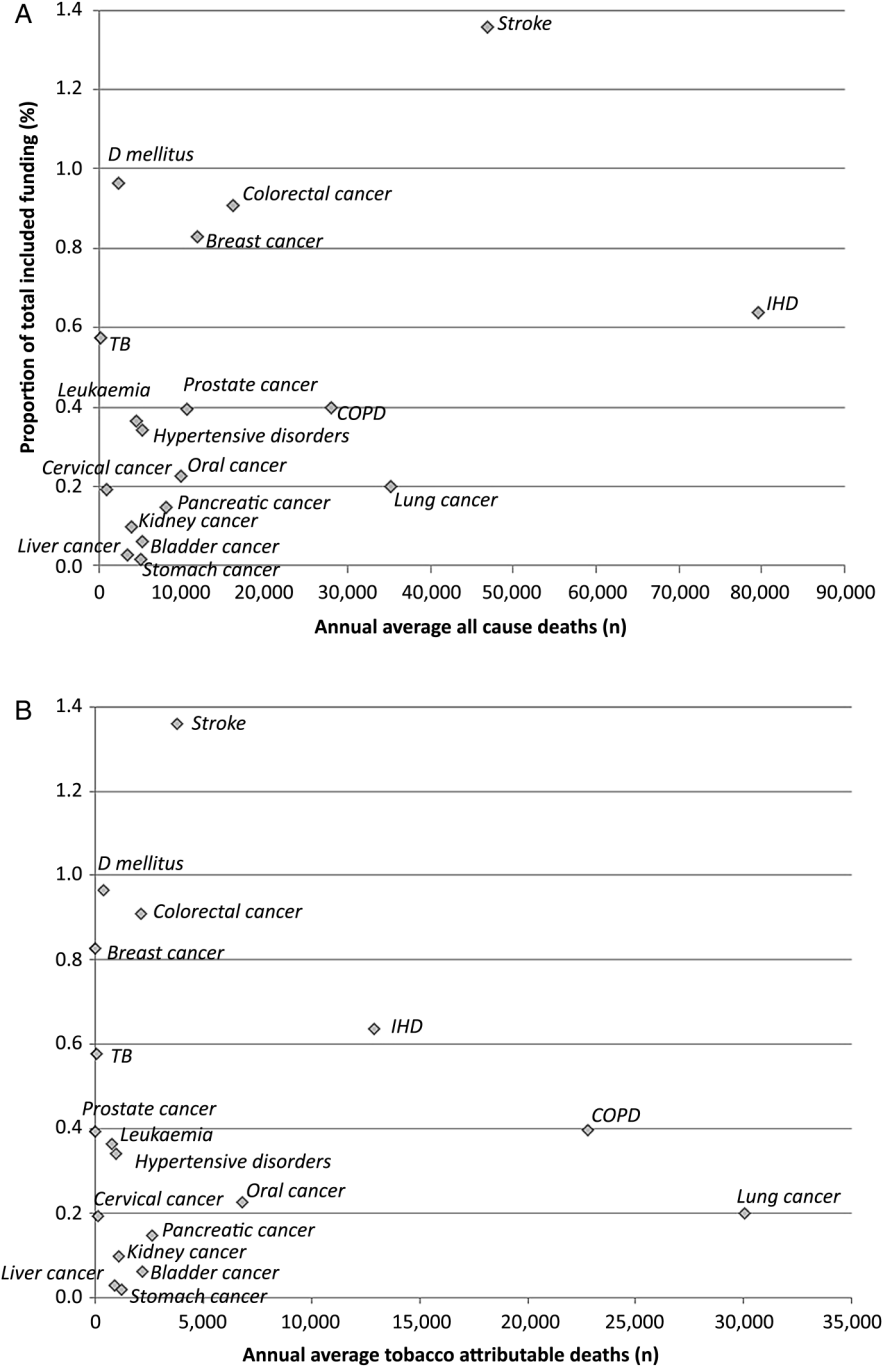
(A) Annual average all cause deaths versus proportion of funding received. (B) Annual average tobacco attributable deaths versus proportion of funding received. COPD, chronic obstructive pulmonary disease; IHD, ischaemic heart disease; TB, tuberculosis.

We used all cause deaths by tobacco-related disease as a measure of overall disease burden to analyse the relation between disease burden and research investment. Linear regression analysis demonstrated a weak and non-significant association (r^2^=0.16, p=0.1) between these variables. IHD had the highest number of deaths but the 5th largest proportion of total annual average research funding, equivalent to 0.64% of total funding, (figure 3A); stroke received the most funding and had the second highest number of deaths. Lung cancer and COPD had the 3rd and 4th highest number of deaths respectively but the 12th and 7th highest proportion of funding (0.2% and 0.4% of total respectively). Stomach, bladder and liver cancer had very low proportions of overall funding (0.02%, 0.06% and 0.03% respectively) and there was a noticeable difference in funding allocation between several diseases such as kidney cancer (0.1%), diabetes mellitus (0.96%) and liver cancer (0.03%) despite their similar annual average number of deaths.

We carried out further analysis to explore the relation between research investment and tobacco attributable deaths (figure 3B). Linear regression analysis showed no evidence of association (r^2^=0.01, p=0.74). Lung cancer and COPD had the highest number of tobacco attributable deaths but ranked 12th and 7th respectively in funding allocation.

Stroke had the fifth highest number of deaths but the highest funding and many diseases with a similar number of tobacco attributable deaths such as liver cancer, stomach cancer or hypertensive diseases had wide differences in funding allocation.

#### Research spending per all-cause and tobacco attributable death

**Figure 4:**
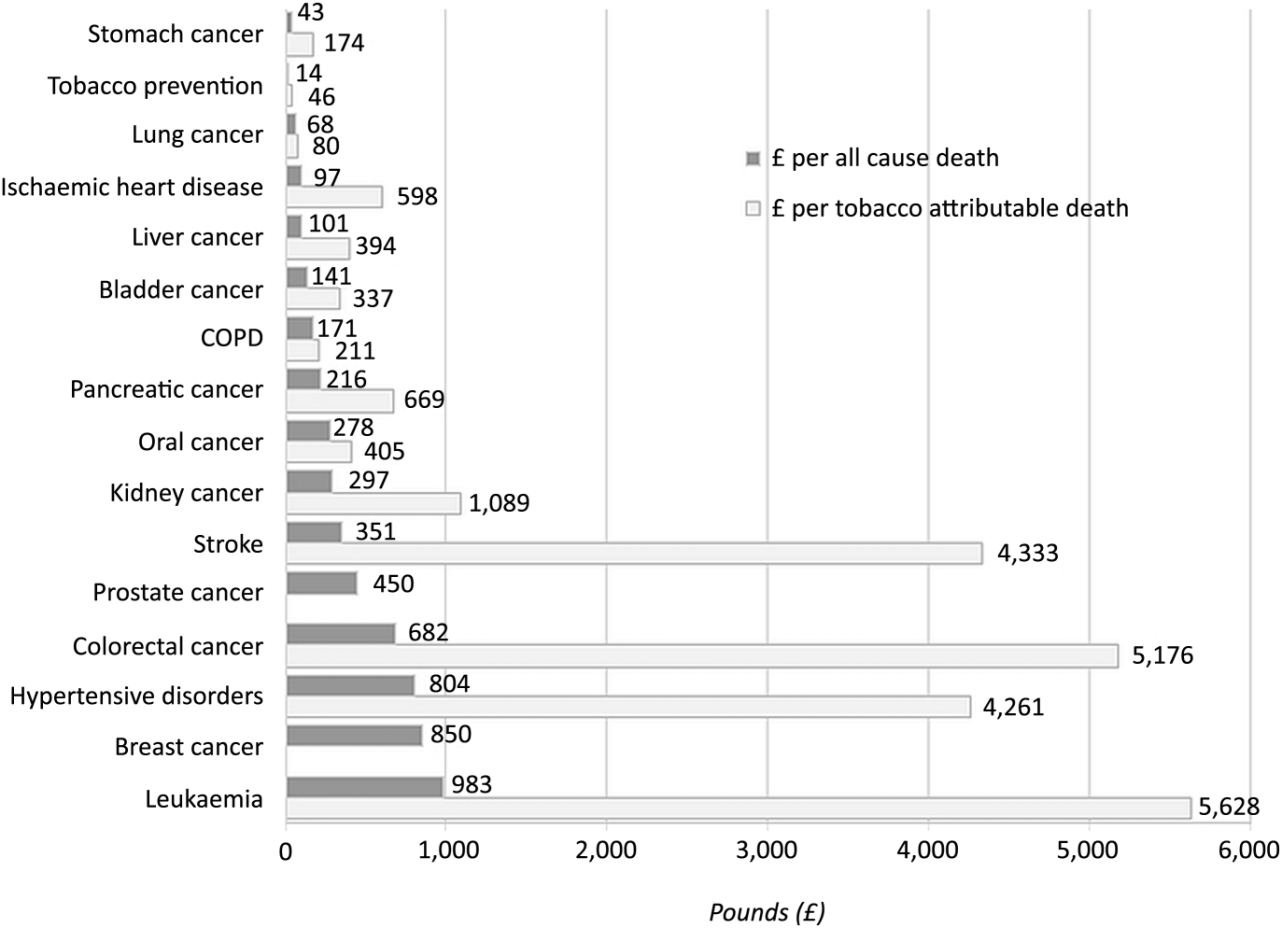
Research spend per death (all-cause and tobacco attributable). COPD, chronic obstructive pulmonary disease.

TB, diabetes mellitus and cervical cancer received the largest amount of funding per all-cause death at £26.8k, £5k and £2.5k, respectively, (not shown in figure 4 due to their relatively large size and effect on the overall scale of the figure). Diseases with a higher number of deaths such as IHD and lung cancer had a relatively small amount of funding per death (£97 and £68, respectively). However, the difference in number of deaths did not account for all differences in funding. Kidney cancer, stomach cancer and hypertensive diseases accounted for similar numbers of deaths but were funded at £297, £43 and £804 per death, respectively, while prostate and oral cancer, with a similar number of deaths were funded at £450 and £278 per death, respectively, (figure 4).

TB, diabetes mellitus and cervical cancer received the largest funding per tobacco-attributable death at £141.2k, £29.3k and £16.3k, respectively, (figures not included in figure 4) and in general those diseases with fewer tobacco attributable deaths had higher spend per attributable death. However, there was again wide variation in funding allocation particularly in diseases with similar numbers of deaths: colorectal cancer received £5k per tobacco attributable death, pancreatic cancer £670 and bladder cancer £340 despite each having around 2000 tobacco attributable deaths per year. The two diseases with the highest number of tobacco attributable deaths, lung cancer and COPD received £80 and £211 per attributable death (figure 4).

Research funding of tobacco prevention amounted to just £46 per death from tobacco-related disease (figure 4), equating to ∼£1 spent researching how to prevent tobacco consumption for every £29 spent researching the consequences of tobacco consumption.

## Discussion

To the best of our knowledge this is the first study to investigate research investment in tobacco-related disease and prevention in the UK. It found that 20% of all deaths each year are from tobacco-related disease, equating to 110 000 people. Over £1.2bn is invested in health research by governmental and charitable bodies each year, £130m of which is on tobacco-related disease and £5m on tobacco prevention or 10.8% and 0.42% of total annual funding respectively. Investment in research into different tobacco-related diseases varies widely from an annual average of £16m for stroke research to £216k for stomach cancer. Investment bears little relation to either overall mortality or tobacco attributable mortality: IHD had the largest number of overall deaths but the fifth highest proportion of research funding, while lung cancer had the highest number of tobacco attributable deaths but the 13th largest proportion of funding. In addition investment in tobacco prevention compared to treatment for tobacco-related disease is relatively low: £46 for every tobacco attributable death spent on tobacco prevention and £29 spent on researching diseases arising from tobacco use for every pound spent on tobacco prevention. It thus appears that research investment into tobacco-related disease and prevention is not associated with overall disease burden as measured by mortality or by degree of disease risk as measured by attributable mortality.

There is a history of relatively low investment into both COPD and lung cancer research.^14^ This has been attributed to both the general public and researcher ‘victim blaming’, ‘victims’ being ill through their own decision to smoke consequently making fund raising more difficult and researchers more reluctant to enter an ‘unpopular’ field of research.^30^ ^31^ Published research shows a higher proportion of studies concerned with tobacco disease treatment rather than addiction^19^ and although studies into lung cancer are now published in journals of a higher than average impact factor,^14^ general tobacco-related studies in the past have been in lower impact journals.^32^ It is not clear why certain diseases are relatively underfunded and there are likely to be a variety of contributory factors including perceptions associated with tobacco research, availability of researchers and availability of appropriate funding. Being able to highlight funding levels, as this study has attempted to do, is thus an important step in attempting to redress any imbalances identified. Mortality is a straightforward, if not comprehensive, measure of disease burden and although not necessarily representative of total disease burden (see below) could usefully be used in any national or international research prioritisation exercise. Studies that have used this measure have shown similar results: in the US a ninefold difference in funding per death of lung cancer compared to breast cancer^33^ and an estimated $1.2k spent on research per death of lung cancer compared to $27k per death of breast cancer,^34^ while in the UK research spending on lung cancer accounted for just 1.4% of all cancer funding compared to 22% of all deaths.^14^ The stark difference between 5-year survival of, for example, breast and lung cancer may not show the impact of research investment difference but does highlight the importance of equity of research investment in relation to need rather than demand.

Alternate methods of measuring disease burden include years of life lost (YLL), disability adjusted life years (DALY), economic burden and prevalence. YLL and DALYs take into account the age at which a person dies and the time spent ill before dying and as such provide a more in-depth measure of societal burden.^35^ We used mortality as a measure of burden of disease because it is simple and unequivocal, but acknowledge that this approach excludes the impact of time spent ill before death or age of death. Comparisons between different measures of burden have shown similarity in funding compared to burden in diseases at either end of the funding spectrum but some differences for those in the ‘middle ground’.^36^ ^37^ Regardless of the metric used, there is a consistency in certain diseases being relatively well funded (breast and prostate cancer, leukaemia) and others relatively poorly funded (lung, bladder and stomach cancer, dementia).^35–39^ Despite its relative simplicity, mortality may well then be a useful starting point while acknowledging that combing this with other measures such as DALYs might provide a more complete picture of funding relative to population disease burden. Using the SIR rather than smoking prevalence estimates to calculate tobacco attributable mortality can over estimate number of deaths,^40^ ^41^ however it is the most commonly used and accepted methodology^42^ and is particularly useful for those countries which do not have valid data on smoking prevalence. The use of tobacco attributable deaths allowed us to assess the relation between level of risk and research investment, and for these comparisons we used the same funding information as for all deaths. Funding information was gained from a broad range of public and charitable bodies that attempted to include all major sources of research funding. However, it is likely that some sources have been missed; in addition commercial and industry funded research is not included in this study given the difficulty in obtaining such data and its lack of direct influence on public policy. Nevertheless such funding may well have an influence on government or charitable research investment not least by highlighting areas of research that are not of a commercially high enough interest to profit making organisations and thus requiring government input. In addition this study did not look at international funding that may have an influence on UK funding and as such does not provide a wider picture of all potentially relevant tobacco-related research investment. This study does nevertheless represent a sizeable portion of UK research funding collected and collated in a comprehensive manner. Classification was carried out by the first author alone, but was carried out a number of times and used the HRCS to ensure consistency. Classification carried out by two organisations corresponded closely to similar classifications carried out by the author. Research funding was allocated to the year of grant award rather than divided across years of award potentially leading to over or under estimation of funding in some years. The primary aim of our research was to compare funding with mortality not to calculate annual trends or to reflect differing annual research priorities. Therefore, despite the obvious skew in annual funding we felt that average annual funding was the most appropriate method with which to compare to average annual deaths.

The UK has the strongest range of tobacco control policies in Europe^43^ and historically has had a strong track record of commitment to evidence-based smoking cessation services.^44^ Primary prevention in general tends to provide excellent value for money^45^ and targeted prevention such as brief interventions to middle-aged smokers can save money.^46^ The continued reduction in smoking prevalence is slowing such that there is now little difference in smoking rates between 2007 and 2012^47^ and it is often the more deprived and vulnerable groups that continue or start to smoke.^48^ ^49^ It could be argued that understanding why people smoke, why they start smoking and helping them to stop would form the spearhead of any research investment. Prevention is a fundamental principle in the NHS 5-Year Forward plan^50^ emphasising the importance of research into preventing smoking uptake, reduction in smoking prevalence and identification of effective tobacco control policies.

There is substantial investment in health research in the UK, yet this covers discrepancies in the amount certain diseases are funded compared to other diseases and to mortality burden. Research into prevention appears to be particularly underfunded. A national research strategy, coordination or system of prioritisation would enable a more robust approach to research investment and create a more equitable investment in different disease areas and of primary prevention and treatment. We would argue that research allocation based on a recognisable and coordinated prioritisation system would allow for substantial investment in areas with the greatest burden. However, it is measured, tobacco use creates the single highest health burden in the UK and investment in research should reflect this burden.
