# Supplementary figures and images for: Research funding for addressing tobacco-related disease: an analysis of UK investment between 2008 and 2012
Source: BMJ Open. 2016 Jul 4;6(7):e011609. doi: 10.1136/bmjopen-2016-011609 (PMC4947797; doi:10.1136/bmjopen-2016-011609)

Supplementary Figure 1

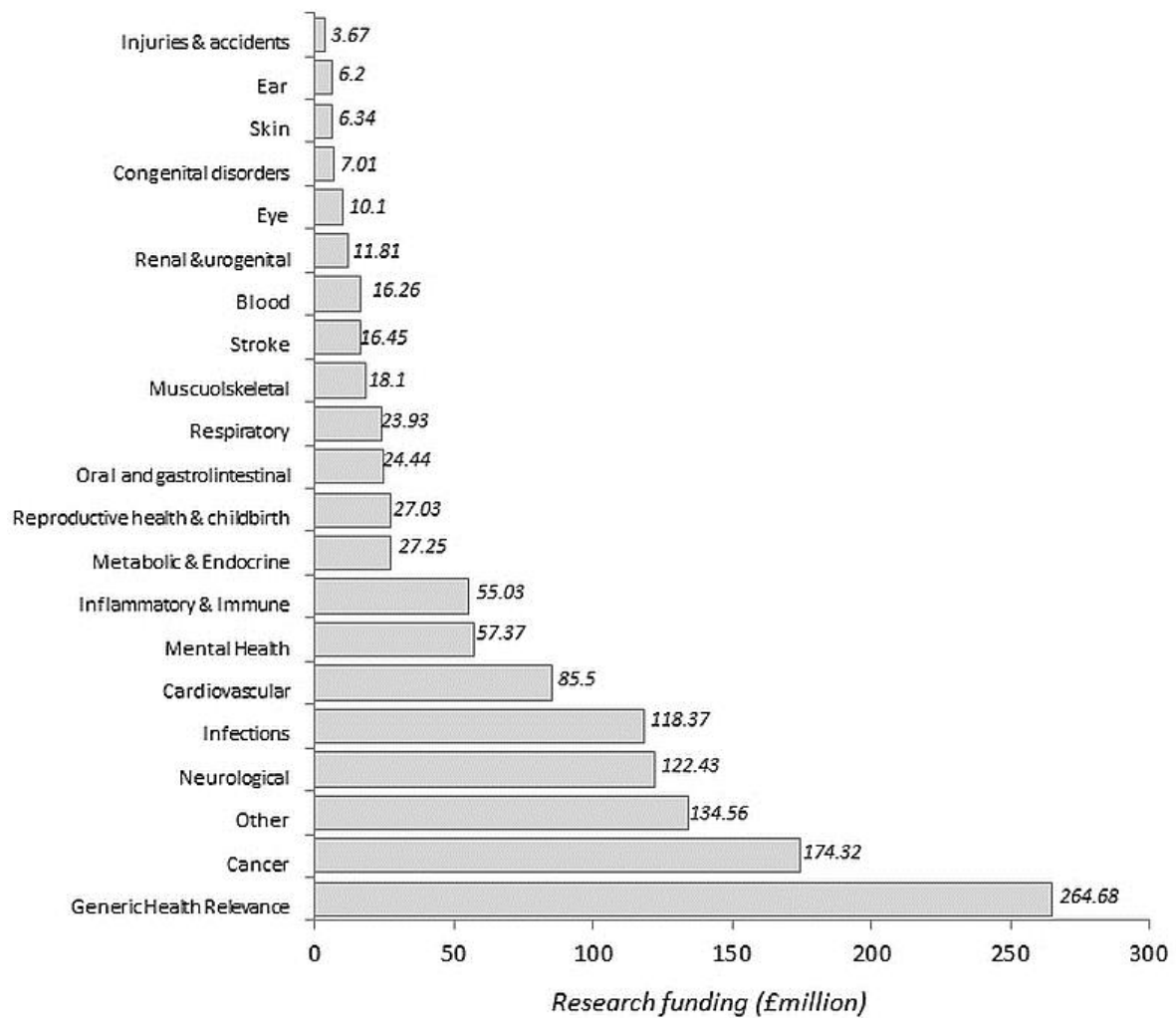

Supplement: Supplementary figure [file bmjopen-2016-011609supp_figure.pdf]
